# The survival benefits of local surgery in stage IV breast cancer are not affected by breast cancer subtypes: a population-based analysis

**DOI:** 10.18632/oncotarget.18889

**Published:** 2017-06-29

**Authors:** San-Gang Wu, Wen-Weng Zhang, Jia-Yuan Sun, Feng-Yan Li, Huan-Xin Lin, Juan Zhou, Zhen-Yu He

**Affiliations:** ^1^ Department of Radiation Oncology, Xiamen Cancer Hospital, The First Affiliated Hospital of Xiamen University, Xiamen 361003, People’s Republic of China; ^2^ Department of Radiation Oncology, Sun Yat-sen University Cancer Center, State Key Laboratory of Oncology in South China, Collaborative Innovation Center of Cancer Medicine, Guangzhou 510060, People’s Republic of China; ^3^ Department of Obstetrics and Gynecology, The First Affiliated Hospital of Xiamen University, Xiamen 361003, People’s Republic of China

**Keywords:** breast cancer, SEER program, surgery, subtype, survival

## Abstract

This retrospective study aimed to investigate the clinical value of local surgery in stage IV BC and determined whether the survival outcomes were affected by the breast cancer subtype (BCS). Women with de novo stage IV BC from 2010 to 2013 were included using the Surveillance Epidemiology and End Results database. Univariate and multivariate Cox regression analyses were performed to evaluate the prognostic factors for breast cancer-specific survival (BCSS) and overall survival (OS). Among 9,256 patients were identified, 3,130 (33.8%) were received local surgery. Patients with hormone receptor (HR)+/human epidermal growth factor receptor 2 (HER2)- subtype were less likely to receive local surgery, while HR-/HER2- tumors were more likely to receive surgery. Multivariate analyses revealed that local surgery improved survival, surgical intervention was an independent favorable prognostic factor for BCSS (P < 0.001) and OS (P < 0.001). Patients who receipt of surgery had better survival outcomes compared with the non-surgery group, and the survival benefits of local surgery were not affected by the BCS status. Local surgery was improved survival for patients with stage IV BC regardless of the BCS status.

## INTRODUCTION

Breast cancer (BC) is the most common malignancy diagnosed in women worldwide [1–4]. The majority of BC patients are diagnosed as having an early disease stage. However, 2.4-6% of patients had metastatic disease at initial presentation, which was associated with poor survival [2, 5, 6]. Patients with de novo metastatic disease have a longer median survival time compare with patients with relapsed BC (39.2 months vs. 27.2 months) [7]. This difference may be due to better responses to systemic therapy for BC patients with de novo stage IV disease, while patients had distant relapse might present with therapeutic resistance. There may also be biological differences between synchronous and metachronous metastases dictated by the induction of resistant clones in tumors [8]. Patients with de novo stage IV BC may present with a particular metastatic subtype, requiring the development of a distinct treatment approach.

Treatment strategies for stage IV breast cancer have significantly advanced in the last two decades due to a better understanding of the heterogeneity of the disease. Treatment approaches include endocrine therapies, targeted therapies, and different types of chemotherapy, which are based upon the identification of breast cancer subtype (BCS) based on the hormone receptor (HR) and human epidermal growth factor receptor 2 (HER2) status [9]. The clinical value of local surgery in stage IV BC remains controversial [10, 11]. An increasing number of studies have found that local treatment including surgery or radiotherapy significantly prolongs the survival in BC patients with de novo stage IV disease [12–18]. However, most of these studies spanned a long period of time, and the value of BCS in predicting the survival of patients treated with surgery is still limited [19, 20]. In the current study, we performed a population-based analysis to evaluate the clinical value of local surgery of BC patients with de novo stage IV disease.

## RESULTS

### Patient characteristics

In total, 9,256 patients diagnosed with stage IV BC from 2010 to 2013 were identified including 3,130 (33.8%) patients received local surgery. The patient characteristics are showed in Table 1. Patients who chose local surgery tended to be younger, larger tumor sizes, poorly/undifferentiated disease, advanced nodal stage, and married. Patients with HR+/HER2- tumors were less likely to received local surgery, while patients with HR-/HER2- tumors were more likely to undergo local surgery. There was no difference for performing surgery among the race groups.

**Table 1:** Patient characteristics

Characteristic	n (%)	No surgery (%)	Surgery (%)	*P value*
Age (years)				
<35	336 (3.6)	189 (3.1)	147 (4.7)	< 0.001
35-50	1926 (20.8)	1129 (18.4)	797 (25.5)	
>50	6994 (75.6)	4808 (78.5)	2186 (69.8)	
Race (n = 9,217)				
White	6936 (75.3)	4603 (75.5)	2333 (74.7)	0.134
Black	1570 (17.0)	1046 (17.2)	524 (16.8)	
Other	711 (7.7)	446 (7.3)	265 (8.5)	
Grade (n = 7,665)				
Well differentiated	569 (7.4)	386 (8.2)	183 (6.2)	< 0.001
Moderately differentiated	3169 (41.3)	2145 (45.4)	1024 (34.8)	
Poorly/undifferentiated	3927 (51.2)	2195 (46.4)	1732 (58.9)	
Tumor size (cm) (n = 7,504)				
≤2	1264 (16.8)	810 (17.6)	454 (15.6)	0.017
>2-5	3567 (47.5)	2193 (47.8)	1374 (47.2)	
>5	2673 (35.6)	1588 (34.6)	1085 (37.2)	
Nodal stage (n = 8,431)				
N0	1893 (22.5)	1365 (25.4)	528 (17.3)	< 0.001
N1	4006 (47.5)	2831 (52.7)	1175 (38.5)	
N2	1094 (13.0)	496 (9.2)	598 (19.6)	
N3	1438 (17.1)	684 (12.7)	754 (24.7)	
Breast cancer subtype				
HR+/HER2-	5566 (60.1)	3837 (62.6)	1729 (55.2)	< 0.001
HR+/HER2+	1532 (16.6)	995 (16.2)	537 (17.2)	
HR-/HER2+	847 (9.2)	527 (8.6)	320 (10.2)	
HR-/HER2-	1311 (14.2)	767 (12.5)	544 (17.4)	
Marital status (n = 8,751)				
Unmarried	4751 (54.3)	3292 (56.9)	1459 (49.1)	< 0.001
Married	4000 (45.7)	2490 (43.1)	1510 (50.9)	

HER2, human epidermal growth factor receptor; HR, hormone receptor.

The patient characteristics according to various BCSs are summarized in Table 2. In HR+/HER2-, HR+/HER2+, and HR-/HER2+ stage IV BC, patients who chose local surgery were younger, had poorly/undifferentiated disease, advanced nodal stage, and married. In HR-/HER2- subtype, patients who received local surgery were more likely to have younger age, poorly/undifferentiated disease, larger tumor size, and advanced nodal stage.

**Table 2:** Patient characteristics according to different breast cancer subtypes

Characteristic	HR+/HER2-			HR+/HER2+			HR-/HER2+			HR-/HER2-		
	No surgery (%)	Surgery (%)	*P value*	No surgery (%)	Surgery (%)	*P value*	No surgery (%)	Surgery (%)	*P value*	No surgery (%)	Surgery (%)	*P value*
Age (years)												
<35	94 (2.4)	52 (3.0)	< 0.001	47 (4.7)	45 (8.4)	< 0.001	21 (4.0)	26 (8.1)	0.009	27 (3.5)	24 (4.4)	0.031
35-50	636 (16.6)	424 (24.5)		216 (21.7)	145 (27.0)		120 (22.8)	86 (26.9)		157 (20.5)	142 (26.1)	
>50	3107 (81.0)	1253 (72.5)		732 (73.6)	347 (64.6)		386 (73.2)	208 (65.0)		583 (76.0)	378 (69.5)	
Race												
White	2977 (78.0)	1346 (78.1)	0.373	735 (74.5)	402 (74.9)	0.373	378 (72.1)	221 (69.5)	0.231	513 (67.0)	364 (67.0)	0.863
Black	562 (14.7)	237 (13.7)		180 (18.2)	89 (16.6)		98 (18.7)	56 (17.6)		206 (26.9)	142 (26.2)	
Other	279 (7.3)	141 (8.2)		72 (7.3)	46 (8.6)		48 (9.2)	41 (12.9)		47 (6.1)	37 (6.8)	
Grade												
Well differentiated	354 (12.2)	169 (10.4)	< 0.001	17 (2.1)	8 (1.6)	< 0.001	1 (0.2)	3 (1.0)	0.042	14 (2.3)	3 (0.6)	< 0.001
Moderately differentiated	1547 (53.5)	733 (45.2)		339 (42.1)	162 (32.1)		123 (29.2)	67 (22.3)		136 (22.3)	62 (12.1)	
Poorly/undifferentiated	989 (34.2)	719 (44.4)		450 (55.8)	334 (66.3)		297 (70.5)	230 (76.7)		459 (75.4)	449 (87.4)	
Tumor size (cm)												
≤2	534 (18.5)	261 (16.0)	0.074	128 (17.1)	82 (16.6)	0.074	50 (13.2)	53 (18.5)	0.164	98 (17.3)	58 (11.6)	0.018
>2-5	1401 (48.4)	794 (48.6)		375 (50.0)	241 (48.9)		175 (46.1)	127 (44.3)		242 (42.7)	212 (42.4)	
>5	959 (33.1)	578 (35.4)		247 (32.9)	170 (34.5)		155 (40.8)	107 (37.3)		227 (40.0)	230 (46.0)	
Nodal stage												
N0	948 (28.5)	290 (17.1)	< 0.001	208 (23.4)	90 (17.2)	< 0.001	76 (16.1)	47 (15.1)	< 0.001	133 (19.3)	101 (19.1)	< 0.001
N1	1721 (51.7)	637 (37.6)		483 (54.5)	210 (40.2)		264 (55.9)	115 (37.0)		363 (52.8)	213 (40.3)	
N2	304 (9.1)	345 (20.4)		93 (10.5)	104 (19.9)		51 (10.8)	65 (20.9)		48 (7.0)	84 (15.9)	
N3	356 (10.7)	420 (24.8)		103 (11.6)	119 (22.8)		81 (17.2)	84 (27.0)		144 (20.9)	131 (24.8)	
Marital status												
Unmarried	2064 (57.1)	800 (48.8)	< 0.001	528 (56.2)	241 (47.1)	< 0.001	274 (55.4)	140 (47.3)	0.028	426 (58.2)	278 (53.2)	0.076
Married	1551 (42.9)	838 (51.2)		412 (43.8)	271 (52.9)		221 (44.6)	156 (52.7)		306 (41.8)	245 (46.8)	

HER2, human epidermal growth factor receptor; HR, hormone receptor.

### Survival

The median follow-up period was 13 months. Among them, there were 3,723 deaths, including 3,239 (87.0%) died with breast cancer related disease. The median breast cancer-specific survival (BCSS) time was 34 months, and the 1, 2, and 3-year BCSS rates were 75.0%, 59.8%, and 47.7%, respectively. The median overall survival (OS) time was 29 months, and the 1, 2, and 3-year OS rates were 71.7%, 55.4%, and 43.0%, respectively.

### Prognostic analysis

**Figure 1:**
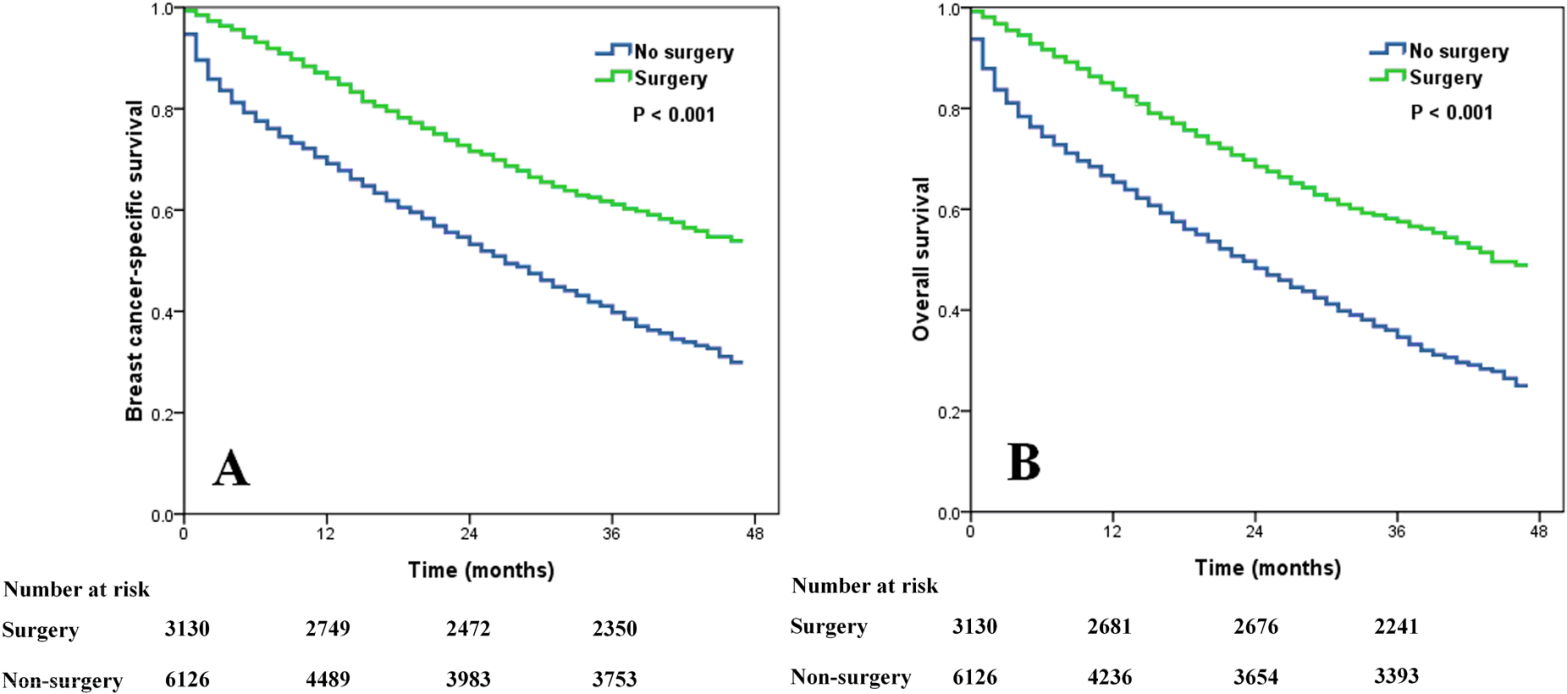
Impact of surgery on breast cancer-specific survival (A) and overall survival (B) in stage IV breast cancer

Univariate and multivariate Cox analysis indicated that local surgery improved survival. Surgical treatment was an independent favorable prognostic factor for BCSS (hazard ratio [HR], 0.451; 95% confidence interval [CI], 0.409-0.498; *P* < 0.001) and OS (HR, 0.457; 95% CI, 0.416-0.501; *P* < 0.001) in the multivariate analysis. The 3-year BCSS was 61.1% and 39.8% in the surgery group and the non-surgery group, respectively. The median BCSS time was significantly increased in the surgery group compared with the non-surgery group; the median BCSS time was not reached in the surgery group and was 27 months in the non-surgery group (log rank *P* < 0.001; Figure 1A). The 3-year OS was 57.5% and 34.6% in the surgery group and the non-surgery group, respectively, and the median OS time was also prolonged (44 months vs. 23 months, log rank *P* < 0.001; Figure 1B). Age, race, grade, tumor size, nodal stage, BCS, and marital status were also significantly associated with BCSS and OS in the multivariate analysis (Table 3 and Table 4).

**Table 3:** Univariate Cox regression analysis of prognostic factors influencing the survival of stage IV breast cancer patients

Characteristic	BCSS			OS		
	HR	95% CI	*P value*	HR	95% CI	*P value*
Age (years)						
<35	1			1		
35-50	1.280	1.003-1.633	0.047	1.320	1.044-1.671	0.021
>50	1.900	1.506-2.398	< 0.001	2.065	1.649-2.585	< 0.001
Race						
White	1			1		
Black	1.279	1.172-1.396	< 0.001	1.295	1.194-1.405	< 0.001
Other	0.848	0.737-0.975	0.021	0.851	0.747-0.969	0.015
Grade						
Well differentiated	1			1		
Moderately differentiated	1.445	1.202-1.738	< 0.001	1.328	1.125-1.566	0.001
Poorly/undifferentiated	2.078	1.735-2.489	< 0.001	1.863	1.585-2.189	< 0.001
Tumor size (cm)						
≤2	1			1		
>2-5	1.042	0.928-1.171	0.487	1.003	0.902-1.116	0.952
>5	1.315	1.168-1.481	< 0.001	1.229	1.102-1.371	< 0.001
Nodal stage						
N0	1			1		
N1	0.946	0.862-1.039	0.246	0.864	0.793-0.941	0.001
N2	0.831	0.731-0.945	0.005	0.777	0.690-0.876	< 0.001
N3	0.920	0.819-1.034	0.164	0.846	0.759-0.943	0.002
Breast cancer subtype						
HR+/HER2-	1			1		
HR+/HER2+	0.856	0.770-0.952	0.004	0.840	0.761-0.928	0.001
HR-/HER2+	1.185	1.047-1.342	0.007	1.187	1.057-1.331	0.004
HR-/HER2-	2.534	2.324-2.763	< 0.001	2.491	2.298-2.701	< 0.001
Marital status						
Unmarried	1			1		
Married	0.701	0.652-0.754	< 0.001	0.678	0.633-0.725	< 0.001
Surgery						
No	1			1		
Yes	0.480	0.443-0.520	< 0.001	0.478	0.444-0.515	< 0.001

BCSS, breast cancer-specific survival; CI, confidence interval; HER2, human epidermal growth factor receptor; HR, hormone receptor; HR, hazard ratio; OS, overall survival.

**Table 4:** Multivariate Cox regression analysis of prognostic factors influencing the survival of stage IV breast cancer patients

Characteristic	BCSS			OS		
	HR	95% CI	*P value*	HR	95% CI	*P value*
Age (years)						
<35	1			1		
35-50	1.193	0.896-1.589	0.227	1.272	0.961-1.684	0.092
>50	1.674	1.274-2.200	< 0.001	1.871	1.432-2.445	< 0.001
Race						
White	1			1		
Black	1.112	0.989-1.251	0.075	1.121	1.004-1.250	0.041
Other	0.858	0.714-1.031	0.102	0.878	0.740-1.042	0.137
Grade						
Well differentiated	1			1		
Moderately differentiated	1.426	1.137-1.787	0.002	1.270	1.040-1.551	0.019
Poorly/undifferentiated	2.032	1.620-2.550	< 0.001	1.774	1.452-2.169	< 0.001
Tumor size (cm)						
≤2	1			1		
>2-5	1.010	0.880-1.158	0.891	0.993	0.875-1.126	0.908
>5	1.220	1.060-1.406	< 0.001	1.166	1.023-1.328	0.021
Nodal stage						
N0	1			1		
N1	0.827	0.735-0.931	0.002	0.752	0.675-0.837	< 0.001
N2	0.881	0.751-1.034	0.120	0.820	0.708-0.951	0.008
N3	0.866	0.746-1.005	0.058	0.793	0.691-0.911	0.001
Breast cancer subtype						
HR+/HER2-	1			1		
HR+/HER2+	0.859	0.746-0.989	0.034	0.855	0.749-0.977	0.021
HR-/HER2+	1.132	0.954-1.342	0.155	1.227	1.050-1.434	0.010
HR-/HER2-	2.618	2.324-2.950	< 0.001	2.602	2.327-2.910	< 0.001
Marital status						
Unmarried	1			1		
Married	0.749	0.682-0.824	< 0.001	0.715	0.654-0.782	< 0.001
Surgery						
No	1			1		
Yes	0.451	0.409-0.498	< 0.001	0.457	0.416-0.501	< 0.001

BCSS, breast cancer-specific survival; CI, confidence interval; HER2, human epidermal growth factor receptor; HR, hormone receptor; HR, hazard ratio; OS, overall survival.

### Effects of surgery on survival according to BCS

**Figure 2:**
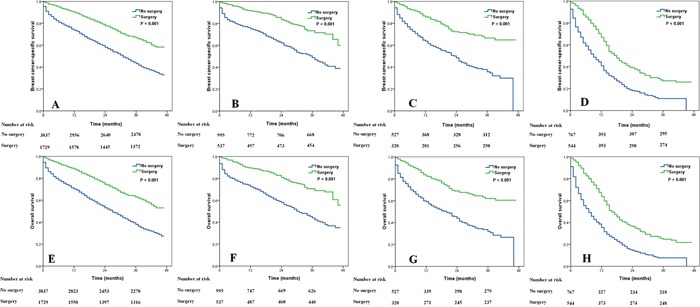
Impact of surgery on breast cancer-specific survival (**A**, HR+/HER2-; **B**, HR+/HER2+; **C**, HR-/HER2+; **D**, HR-/HER2-) and overall survival (**E**, HR+/HER2-; **F**, HR+/HER2+; **G**, HR-/HER2+; **H**, HR-/HER2-) in stage IV breast cancer according to different breast cancer subtypes.

The clinical value of surgery on survival based on BCS were examined. The results showed that those who received surgery also had better BCSS and OS compared with the non-surgery group regardless of the BCS status (all *P* < 0.001) (Figure 2).

## DISCUSSION

Using a population-based analysis from the Surveillance Epidemiology and End Results (SEER) database, we sought to evaluate the role of surgical treatment for the intact primary breast tumor in de novo stage IV BC patients. We further assessed the clinical value of surgery in patients with various BCS. Our results indicated that patients who receipt of surgery was associated with better survival, and the survival benefits of local surgery were not affected by BCS.

Currently, the role of local treatment for stage IV BC is still controversial. In a multicenter prospective registry study of 112 stage IV BC patients, the 3-year OS were 77% and 76% (*P* = 0.85), and the median OS time were 77 and 71 months in patients with and without local surgery (*P* = 0.85), respectively [21]. However, in a randomized prospective Turkish study of 274 stage IV BC, at median 40 months follow-up, the surgery group had statistically significant improvement in median survival compared to the systemic therapy group (46 months vs. 37 months, *P* = 0.005), for a 9-month overall advantage [22]. In addition, an increasing number of retrospective studies support the clinical value of surgery in stage IV BC [14–18, 23, 24]. In our study, patients who received local surgery appear to have a better survival. We hypothesize, along with others that local surgery could improve survival outcomes by providing locoregional control, eliminating potential seed sources, possibly a stimulant of metastatic disease sites, and potentially modulate the immune response [25, 26].

BCS according to HR and HER2 status are widely used to evaluate prognosis, predict treatment effects and guide treatment. Results from a meta-analysis have shown that there were no statistically significant differences on survival between patients with and without local surgery with regards to HR status in BC patients with stage IV disease [15]. In our study, fewer HR+/HER2- patients were received surgery, while patients with HR-/HER2- tumors were more likely to undergo surgery. The main reason for this difference is unclear. Our survival analysis showed that the survival of patient with HR+/HER2+ tumors was improved compared with HR+/HER2- subtype patients, while patients with HR-/HER2- subtype had the worst survival. Therefore, it can be assumed that there are more systemic treatment strategies available for HR positive and HER2 positive patients, whose long-term survivals are significantly superior to those of HR+/HER2- and HR-/HER2- subtype patients. However, studies based on neoadjuvant therapy have found that there were relatively higher rates of pathological complete response in HR-/HER2- patients [27, 28], which would likely influence the choice of surgery in stage IV BC.

Studies focusing on the role of BCS on surgical outcome in BC patients with stage IV disease are limited. A study by Chen *et al*. found that local treatment including surgery or radiotherapy improved survival in patients with HR+/HER2- (*P* = 0.0001) and HR±/HER2+ subtypes (*P* = 0.0012), but survival did not improve in HR-/HER2- tumors after local treatment (*P* = 0.9575) [19]. Neuman *et al*. also found that local surgery was associated with better survival in patients with HR+ or HER2+ disease (*P* = 0.004), but was not associated with improved survival in HR-/HER2- disease, where nearly 90% of patients who were eligible for endocrine therapy or trastuzumab related therapy [20]. The Turkish study also found that local surgery significantly improved OS in HR+ (*P* = 0.01) disease and HER2- disease (*P* = 0.01) compared with the non-surgery group [22]. However, there was no survival benefit of surgery in retrospective or prospective randomized studies according to HR and HER2 status [21, 23, 24]. The number of HR-/HER2- patients in the studies by Chen *et al*. and Neuman *et al*. was only 45 and 35, respectively [19, 20]. Therefore, we cannot conclusively establish the value of surgical intervention for HR-/HER2- patients. In our study, we identified 9,256 patients including 1,311 patients with HR-/HER2-. Our results found that surgical intervention improved survival regardless of the BCS status. The survival benefit was greatest in HR+/HER2-, HR+/HER2+, and HR-/HER2+ subtypes, while HR-/HER2- patients also experienced a significantly improved survival (24.7% vs. 7.7%).

With progress in early detection and comprehensive treatment, the survival outcomes of stage IV BC patients have shown a gradual increase. A similar trend in increased survival was also observed in patients undergoing surgery, with a 3-year OS of approximately 50% in patients undergoing surgery between the years 2006 and 2009 [16]. The 3-year OS reached 57.5% in the surgery group in our study, which included patients diagnosed between 2010 and 2013. In a study by Badwe and colleagues, no HER2+ patients in the non-surgery group received targeted therapy, while only 2% of HER2+ patients underwent targeted therapy in the surgery group [23]. In a study by the National Comprehensive Cancer Network Breast Cancer Outcomes Database, 42% and 30% of HER2+ patients in the non-surgery group and the surgery group received targeted therapy, respectively. However, the enrolled patients were receipt local surgery and followed by systemic therapy, therefore the therapeutic effect of systemic therapy was unclear [24]. In the study by Neuman *et al*., most HR+ and HER2+ patients received the corresponding targeted therapy, and the results indicated that surgery improved the survival of HR+ and HER2+ patients [20]. Anti-HER2 therapy is standard treatment approach in the United States and influences survival [29]. However, due to the limitations of the SEER database, we could not determine the sequential order of surgery and systemic therapy, chemotherapy regimes, targeted therapy and endocrine therapy. In the era of precision medicine, it is possible to establish the treatment regimen for stage IV breast cancer for each patient based on genetic and biological markers. With the progress in comprehensive treatments, there are more therapeutic regimens available for advanced breast cancer to prolong survival and local surgical treatment may provide additional benefits.

Our study also found that the probability of unmarried patients undergoing surgery was significantly lower than married patients, and multivariate analysis results showed that there were significant differences in survival outcomes based on marital status, married patients had better CSS and OS compared to unmarried patients. Severe psychological and socioeconomic stress have been proposed to contribute to the breast cancer diagnosis and being unmarried was significantly associated with serious psychological distress among breast cancer patients [30]. Previous studies have found that unmarried status was an important predictor of outright refusal of surgery and radiation, early discontinuation, and non-adherence to adjuvant therapy [31, 32]. In addition, the complex multimodal treatment of stage IV BC requires intense psychosocial support. Therefore, marriage may be having a protective effect on survival in stage IV BC. We need to acknowledge several limitations in our study. First, retrospective studies have an inherent bias. Second, the SEER database lacks information on the curative effect evaluation after corresponding systemic therapy. Third, the SEER database also lacks information on the specific type of systemic therapy, targeted therapy and endocrine therapy, the sequential order and specific indications of surgery and systemic therapy. In addition, the median follow up was only 13 months, this may due to that the BCS was started collecting in SEER after 2010. Therefore, long-term follow-up is an important need for the further survival analysis.

In conclusion, according to our results, local surgery was associated with better survival for BC patients with stage IV disease regardless of the BCS status. However, the study is a retrospective observational study and selection bias can not be excluded. Further randomized clinical trials will be essential to understanding our observed association between the receipt of local surgery and improved survival outcomes.

## MATERIALS AND METHODS

### Patients

Female patients diagnosed with stage IV BC from 2010 to 2013 were included using the SEER program [33]. Patients were identified if they met the following inclusion criteria: 1) stage IV BC at initial presentation; 2) BC as the primary cancer diagnosis; 3) local treatment strategies including surgery (mastectomy or breast-conserving surgery) or non-surgery were available; 4) complete results of estrogen receptor, progesterone receptor, and HER2 status. This study was based on the public-use data from the SEER program and we have got permission to access the database (reference number: 10269-Nov2015). This study was approved by the ethics committee of the Xiamen Cancer Hospital, the First Affiliated Hospital of Xiamen University and Sun Yat-sen University Cancer Center.

### Demographic and clinicopathological features

The demographic and clinicopathological characteristics were collected as follows: age, race, tumor size, tumor grade, lymph node status, HR status, HER2 status, marital status, and local treatment. The BCS status were started collecting after 2010 in SEER database, which defined as four major subtypes as follows: HR+/HER2-, HR+/HER2+, HR-/HER2+ and HR-/HER2-. The primary study endpoints of this study were BCSS and OS.

### Statistical analysis

The qualitative parameters were compared between the different subgroups using the exact chi-square test and Fisher’s exact probability tests. Survival rates were calculated using the Kaplan-Meier method and compared using the log-rank test. Univariate and multivariate Cox regression analyses were used to determine the risk factors for BCSS and OS. All statistical analyses were performed using the SPSS statistical software package (version 20.0; IBM Corporation, Armonk, NY, USA). A *P*-value < 0.05 was considered to be statistically significant in all analyses.

## References

[1] Cancer Research UK (2014). Worldwide cancer statistics. Cancer Res UK.

[2] Siegel RL, Miller KD, Jemal A (2016). Cancer statistics, 2016. CA Cancer J Clin.

[3] Zheng R, Zeng H, Zhang S, Chen T, Chen W (2016). National estimates of cancer prevalence in China, 2011. Cancer Lett.

[4] DeSantis CE, Fedewa SA, Goding Sauer A, Kramer JL, Smith RA, Jemal A (2016). Breast cancer statistics, 2015: convergence of incidence rates between black and white women. CA Cancer J Clin.

[5] Jung KW, Won YJ, Kong HJ, Oh CM, Shin A, Lee JS (2013). Survival of korean adult cancer patients by stage at diagnosis, 2006-2010: national cancer registry study. Cancer Res Treat.

[6] Li J, Zhang BN, Fan JH, Pang Y, Zhang P, Wang SL, Zheng S, Zhang B, Yang HJ, Xie XM, Tang ZH, Li H, Li JY (2011). A nation-wide multicenter 10-year (1999-2008) retrospective clinical epidemiological study of female breast cancer in China. BMC Cancer.

[7] Dawood S, Broglio K, Ensor J, Hortobagyi GN, Giordano SH (2010). Survival differences among women with de novo stage IV and relapsed breast cancer. Ann Oncol.

[8] Senkus E (2014). Synchronous and metachronous metastatic breast cancer—two incarnations of the same beast?. Breast.

[9] Cadoo KA, Traina TA, King TA (2013). Advances in molecular and clinical subtyping of breast cancer and their implications for therapy. Surg Oncol Clin N Am.

[10] Khan SA (2013). Surgery for the intact primary and stage IV breast cancer…lacking “robust evidence”. Ann Surg Oncol.

[11] Khan SA (2016). Surgical management of de novo stage IV breast cancer. Semin Radiat Oncol.

[12] Nguyen DH, Truong PT, Alexander C, Walter CV, Hayashi E, Christie J, Lesperance M (2012). Can locoregional treatment of the primary tumor improve outcomes for women with stage IV breast cancer at diagnosis?. Int J Radiat Oncol Biol Phys.

[13] Le Scodan R, Stevens D, Brain E, Floiras JL, Cohen-Solal C, De La Lande B, Tubiana-Hulin M, Yacoub S, Gutierrez M, Ali D, Gardner M, Moisson P, Villette S (2009). Breast cancer with synchronous metastases: survival impact of exclusive locoregional radiotherapy. J Clin Oncol.

[14] Petrelli F, Barni S (2012). Surgery of primary tumors in stage IV breast cancer: an updated meta-analysis of published studies with meta-regression. Med Oncol.

[15] Harris E, Barry M, Kell MR (2013). Meta-analysis to determine if surgical resection of the primary tumour in the setting of stage IV breast cancer impacts on survival. Ann Surg Oncol.

[16] Warschkow R, Güller U, Tarantino I, Cerny T, Schmied BM, Thuerlimann B, Joerger M (2016). Improved survival after primary tumor surgery in metastatic breast cancer: a propensity-adjusted, population-based SEER trend analysis. Ann Surg.

[17] Thomas A, Khan SA, Chrischilles EA, Schroeder MC (2016). Initial surgery and survival in stage IV breast cancer in the United States, 1988-2011. JAMA Surg.

[18] Quinn EM, Kealy R, O'Meara S, Whelan M, Ennis R, Malone C, McLaughlin R, Kerin MJ, Sweeney KJ (2015). Is there a role for locoregional surgery in stage IV breast cancer?. Breast.

[19] Chen PY, Cheng SH, Hung CF, Yu BL, Chen CM (2013). Locoregional therapy in luminal-like and HER2-enriched patients with de novo stage IV breast cancer. Springerplus.

[20] Neuman HB, Morrogh M, Gonen M, Van Zee KJ, Morrow M, King TA (2010). Stage IV breast cancer in the era of targeted therapy: does surgery of the primary tumor matter?. Cancer.

[21] King TA, Lyman J, Gonen M, Reyes S, Hwang ES, Rugo HS, Liu MC, Boughey JC, Jacobs LK, McGuire KP, Storniolo AM, Isaacs C, Meszoely IM (2016). A prospective analysis of surgery and survival in stage IV breast cancer (TBCRC 013). J Clin Oncol.

[22] Soran A, Ozmen V, Ozbas S, Karanlik H, Muslumanoglu M, Igci A, Canturk Z, Utkan Z, Ozaslan C, Evrensel T, Uras C, Aksaz E, Soyder A (2016). A randomized controlled trial evaluating resection of the primary breast tumor in women presenting with de novo stage IV breast cancer: Turkish Study (Protocol MF07-01). J Clin Oncol.

[23] Badwe R, Hawaldar R, Nair N, Kaushik R, Parmar V, Siddique S, Budrukkar A, Mittra I, Gupta S (2015). Locoregional treatment versus no treatment of the primary tumour in metastatic breast cancer: an open-label randomised controlled trial. Lancet Oncol.

[24] Dominici L, Najita J, Hughes M, Niland J, Marcom P, Wong YN, Carter B, Javid S, Edge S, Burstein H, Golshan M (2011). Surgery of the primary tumor does not improve survival in stage IV breast cancer. Breast Cancer Res Treat.

[25] Gnerlich J, Jeffe DB, Deshpande AD, Beers C, Zander C, Margenthaler JA (2007). Surgical removal of the primary tumor increases overall survival in patients with metastatic breast cancer: analysis of the 1988-2003 SEER data. Ann Surg Oncol.

[26] Danna EA, Sinha P, Gilbert M, Clements VK, Pulaski BA, Ostrand-Rosenberg S (2004). Surgical removal of primary tumor reverses tumor-induced immunosuppression despite the presence of metastatic disease. Cancer Res.

[27] Rapiti E, Verkooijen HM, Vlastos G, Fioretta G, Neyroud-Caspar I, Sappino AP, Chappuis PO, Bouchardy C (2006). Complete excision of primary breast tumor improves survival of patients with metastatic breast cancer at diagnosis. J Clin Oncol.

[28] Liedtke C, Mazouni C, Hess KR, André F, Tordai A, Mejia JA, Symmans WF, Gonzalez-Angulo AM, Hennessy B, Green M, Cristofanilli M, Hortobagyi GN, Pusztai L (2008). Response to neoadjuvant therapy and long-term survival in patients with triple-negative breast cancer. J Clin Oncol.

[29] Newman LA (2016). Surgery for stage IV breast cancer: domestic and international disparities. JAMA Surg.

[30] Kaiser NC, Hartoonian N, Owen JE (2010). Toward a cancer-specific model of psychological distress: population data from the 2003-2005 National Health Interview Surveys. J Cancer Surviv.

[31] Aizer AA, Chen MH, Parekh A, Choueiri TK, Hoffman KE, Kim SP, Martin NE, Hu JC, Trinh QD, Nguyen PL (2014). Refusal of curative radiation therapy and surgery among patients with cancer. Int J Radiat Oncol Biol Phys.

[32] Hershman DL, Kushi LH, Shao T, Buono D, Kershenbaum A, Tsai WY, Fehrenbacher L, Gomez SL, Miles S, Neugut AI (2010). Early discontinuation and nonadherence to adjuvant hormonal therapy in a cohort of 8,769 early-stage breast cancer patients. J Clin Oncol.

[33] Surveillance, Epidemiology, and End Results (SEER) Program (www.seer.cancer.gov) SEER*Stat Database: Incidence - SEER 9 Regs Research Data, Nov 2015 Sub (1973-2013) Population Adjustment> - Linked To County Attributes - Total U.S., 1969-2014 Counties, National Cancer Institute, DCCPS, Surveillance Research Program, Surveillance Systems Branch, released April 2016, based on the November 2015 submission

